# Nonverbal Behaviors “Speak” Relational Messages of Dominance, Trust, and Composure

**DOI:** 10.3389/fpsyg.2021.624177

**Published:** 2021-01-26

**Authors:** Judee K. Burgoon, Xinran Wang, Xunyu Chen, Steven J. Pentland, Norah E. Dunbar

**Affiliations:** ^1^Center for the Management of Information, The University of Arizona, Tucson, AZ, United States; ^2^Information Technology and Supply Chain Management, Boise State University, Boise, ID, United States; ^3^Department of Communication, UC Santa Barbara, Santa Barbara, CA, United States

**Keywords:** nonverbal communication, relational messages, dominance, nervousness, trust, deception

## Abstract

Nonverbal signals color the meanings of interpersonal relationships. Humans rely on facial, head, postural, and vocal signals to express relational messages along continua. Three of relevance are dominance-submission, composure-nervousness and trust-distrust. Machine learning and new automated analysis tools are making possible a deeper understanding of the dynamics of relational communication. These are explored in the context of group interactions during a game entailing deception. The “messiness” of studying communication under naturalistic conditions creates many measurement and design obstacles that are discussed here. Possibilities for their mitigation are considered.

## Introduction

A mainstay of interpersonal communication is the concept of relational communication, constituted through a constellation of dimensions along which actors express implicit messages about how they regard one another and their interpersonal relationship. These messages are expressed predominantly through nonverbal rather than verbal signals. Although Burgoon and Hale (1984) have identified up to 12 non-orthogonal themes or dimensions along which relational messages can be exchanged, three of the most prominent ones are dominance, trust, and composure. Until recently, the subtlety with which these messages are sent and received has challenged the ability of scientists to capture and describe them. Human observational skills are subjective and operate at a macroscopic level that constrains the measurement of such messages. Moreover, the laborious nature of manual behavioral coding has been a limiting factor on their use in discerning complex social dynamics. Now, with the benefit of new technologies and methods, the nonverbal means by which humans “speak” relational messages can be uncovered objectively, microscopically and dynamically, sometimes to the point of measurement outstripping our clear understanding but at least prompting intriguing possibilities.

Laboratory studies of human behavior are often critiqued for being artificial and highly scripted, with confederates following strict interview protocols and engaging in unnaturally brief interactions (see, e.g., Frank et al., 2008; Fuller et al., 2011; Frank and Svetieva, 2012). In this paper, we report the results of an experiment in which interactions unfold naturally rather than being scripted, the experimental induction introduces enough range in sentiment for participants to develop favorable and unfavorable judgments of one another, interactions are lengthy enough to produce changes in sentiments, and relational messages are measured at multiple intervals so that their dynamics can be captured over time. Moreover, the methods afford measurement of a wealth of nonverbal signals from the head, face, torso and voice as predictors of participants’ own understanding of the relational messages they are receiving from fellow participants. This permits us to identify the nonverbal signals most likely to express three relational dimensions of interest here–dominance, composure and trust–as interactions progress.

## Background

The concept of relational messages can be traced to the term “metacommunication,” coined by Bateson (1951, 1958) to describe signals that distinguish between the “report” and “command” functions of communication and create a frame for understanding it. The report level refers to the content, whereas the command level directs the recipient, the signaler, or both, how to interpret the verbal content. Usually, the metacommunication is considered the nonverbal signals that accompany the verbal content and serves to clarify, amplify or even contradict the verbal content. This distinction was applied in the clinical context, where Watzlawick et al. (1967) used it to refer to observations of how patients interact with their therapists. Their body language in particular expressed implicit messages of how the patients regarded the therapists. These implicit messages, known as relational communication, became a mainstay of interpersonal communication.

Early work applied the construct in such other contexts as a theory of personality (Leary, 1957), the dimensions of meaning in language (Osgood et al., 1957), interpersonal needs (Schutz, 1966), source credibility (McCroskey, 1966), group decision-making (Bales, 1968), immediacy (Mehrabian, 1971), categories of social relationships (Mehrabian and Ksionzky, 1972), intraspecific displays (Andrew, 1972), transactional social relationships (Millar and Rogers, 1976), and interpersonal interaction (Rogers and Farace, 1975; Parks, 1977) and relationship terms (Knapp et al., 1980). Based on a review of these various literatures, Burgoon and Hale (1984) expanded on the concept to 12 topoi, or generic themes, of relational communication *continua*. The dimensions that emerged as most central and recurrent were dominance-submission and affection-hostility. Additional dimensions included trust and composure. Given their relevance to interpersonal and group communication, these dimensions were chosen to reflect participant judgments in an experiment on group communication. The investigation had as a central focus how deception is enacted in group deliberations, making the topoi of dominance, trust and composure particularly germane. Because exploring the dynamics of relational messaging was additionally one of the objectives of the investigation, and it was thought that affection-hostility (liking) would be unlikely to change over an hour’s discussion, affection-hostility was only measured at the end of the discussion.

## Overview

The experiment examined relational communication and deception over multiple phases during group interaction. The sample was multicultural. The exploration of group interaction across multiple, diverse cultures represents a rare approach in several respects. It examines actual nonverbal behavior as opposed to imagined behavior or self-reports of recollected behavior. It allows lengthy rather than brief interchanges and group rather than dyadic interactions. As well, its inclusion of samples from multiple, diverse cultures is also an improvement over studies that make comparisons between two countries chosen for convenience’ sake, or comparisons by countries rather than self-defined by cultural orientations (see, e.g., Giles et al., in press). The inclusion of samples from eight different locations and six different countries with diverse self-reported cultural orientations adds significant range to the cultures that are represented. All of these characteristics—actual interactions, lengthy interactions, group deliberations, and cultural comparisons across multiple cultural orientations—represent advances in deception and relational communication research. Here we present that portion of the research concerned with the nonverbal features associated with relational communication.

Seldom have the nonverbal behaviors associated with relational communication dimensions been studied in depth because of the laborious nature of manually coding nonverbal behavior (for an exception, see Burgoon and Le Poire, 1999, which was a 3-year undertaking). The current project represents a significant advance into the behavioral particulars and dynamics inherent in nonverbal relational message exchange. The nonverbal behaviors were measured using automated tools and analyses incorporating artificial intelligence. Not only did these measurement and analysis tools make it possible to measure far more behaviors in far less time than with manual coding but made it possible to measure microscopic behavior that is neither measurable by human observers nor observable with the naked eye. It was also possible to record analyses over a longer period of time so as to capture the dynamics of those nonverbal behaviors that are not static. In the current case, we recorded group interactions 1 h in length.

## Hypotheses

### Dominance

Dominance-submission is one of the most fundamental and widely recognized dimensions of human relations (Massey-Abernathy and Haseltine, 2019). Though dominance can be defined from different disciplinary perspectives, we adopt the definition proposed by Burgoon et al. (1998) that interpersonal dominance is “a relational, behavioral, and interactional state that reflects the actual achievement of influence or control over another via communication actions.” This definition indicates that unlike power, which entails potentialities for exerting influence on others, dominance is accomplished through actual dyadic interaction. It is achieved behaviorally through particular interaction strategies, such as threat, elevation or initiation.

Nonverbal behaviors associated with perceived dominance are multi-faceted and vary according to the context. For example, silence can be a symbol of threat and dominance (Bruneau, 1973) in one case while an embodiment of submission in another case. Previous studies have reported that facial expressions, such as lowered brows or a non-smiling mouth, are associated with perceived dominance (Keating et al., 1981; Witkower et al., 2020). On the opposite side, body collapse and gaze avoidance correlate with submissiveness (Weeks et al., 2011). On the vocal side, lower pitch (Cheng et al., 2016), loudness (Tusing and Dillard, 2000), vocal variability, rapid speech rate (Hall et al., 2005), jitter, shimmer, and pleasing voice quality (Hughes et al., 2014) have also been reported to correlate with perceived dominance. Tusing and Dillard (2000) also suggested a gender differentiation in vocal indicators of dominance. Bente et al. (2010) reported *trans*-cultural universalities in the recognition of interpersonal dominance.

Burgoon and Dunbar (2006) categorized three overarching principles associated with the nonverbal expressions of dominance: physical potency, resource control and interaction control, each of which has certain nonverbal manifestations. This set of principles delineates dominance-establishing strategies on a higher level, and based on this taxonomy, we can hypothesize the nonverbal behaviors that might influence perceived dominance. Many of these strategies such as threat or elevation would be inappropriate for ostensibly cooperative group settings. However, others, such as signals of potency through intimidating facial expression, dynamic facial expression and loud voices, indicators of size through gesture and posture, and control of interaction through turn initiation, speaker interruptions and control of gaze patterns would (see Burgoon et al., 2002). Thus, we hypothesize that dominant group members, compared to nondominant members, will have:

• More expansive and upright postures and head positions,
• More gaze and receipt of gaze,
• Less smiling but more expressive facial expressions,
• More initiations of turns-at-talk and longer turns at talk,
• Louder voices, and
• More interruptions of others.

A fuller delineation of dominance signals appears in Table 1, hypothesized relationships between perceived dominance and nonverbal behaviors. Their opposites would signify non-dominance and submission. So, for example, non-dominant group members would have fewer and shorter speaking turns, quieter voices, few interruptions, more smiling, more constricted postures, more head tilt, more rigid facial expressions and more eye gaze while listening than speaking.

**TABLE 1:** Hypothesized relationships between perceived dominance and nonverbal behaviors.

Principles	Strategies	Hypothesized nonverbal signals of dominance
Physical potency	Threat	• More glare and stare
	Size or strength	• Louder voice • Deep-pitched voice • Clear articulation (higher voice quality) • Non-smiling face • Upright head and posture
	Expressivity	• More facial expression • More variation in pitch • More head/body movement • More rapid speaking tempo
Resource control	Command of space	• More open body position • More expansive posture
	Precedence	• Initiation of more turns at talk • Longer turns at talk
	Prerogative	• Choice of seating position
	Possession of valued commodities	• More turns-at-talk • Longer turns at talk
Interactional control	Centrality	• More looking while speaking, less looking while listening • Interruption of others’ speaking turns
	Elevation	• Standing or seating above others
	Initiation	• Initiating a conversation
	Non-reciprocation	• Non-matching of others’ behavior
	Task performance cues	• Self-nomination

To the extent that they can be measured automatically, each of the behaviors in Table 1: Hypothesized relationships between perceived dominance and nonverbal behaviors, can be hypothesized as indicators of dominance. Ones such as elevation, choice of seating position and self-nomination that would not be involved are omitted. The measurements are described in the “Materials and Methods” Section.

### Composure

In the context of relational communication, composure is the degree of tension or relaxation experienced within a relationship. Generally, increased levels of composure during interactions leads to more positive outcomes. For instance, manager composure leads to increased employee satisfaction, motivation, and organizational commitment (Mikkelson et al., 2017). Further, professionals may attempt to present themselves as composed in order to instill trust and confidence (Finch, 2005). In a comprehensive analysis of the nonverbal behaviors that influence perceptions of composure, Burgoon and Le Poire (1999) found indicators associated with pleasantness or positivity, expressivity, involvement and immediacy, relaxation, and conversational management. This suggests that composed individuals are active and engaged communicative partners, while also creating an atmosphere of pleasant relaxation and accessibility for the interaction. Not all of these factors like conversational management are as easily exhibited in groups as in dyads, and proxemic immediacy toward one group member might mean non-immediacy with another, but most are relevant in the group context. Thus, the composed group member should show pleasant facial and vocal emotion, expressive (varied) faces and voices, high amount of talk time, relaxed posture, relaxed face and head, relaxed voice, deep pitch, relaxed laughter, moderate loudness and moderate tempo. On the other hand, lower duration of eye contact, non-smiling mouth movement and more jittery hand movement may be signals of anxiety (Waxer, 1977).

Regarding vocal activity, Daly et al. (1977) found that frequency and duration of interactions was positively related to composure, although ratings of composure leveled off and declined as individuals’ vocal activity surpassed 50–60 percent of total time in groups. Presumably, composed individuals must balance between being active and engaged but also preserving a degree of comfort that requires some holding back. Excessive vocalization may undermine the impression of composure as individuals walk the line between being engaged but not overwhelmingly so.

At the other end of the composure continuum, nervousness and communication avoidance or apprehensiveness may also lessen composure evaluations (Burgoon and Koper, 1984). Whereas composure can instill confidence and success, reticent communication is seen as communicating disinterest or social anxiety (Burgoon and Hale, 1983). Reticent communicators are described as being disengaged from a conversation, withdrawing or attempting to avoid interaction altogether. This can occur for a variety of reasons including chronic communication apprehension or situational attempts to suppress information exchange. Reticence can lead to suspicion and stalemate.

Perceptions of reticent communicators arise from nonverbal behaviors associated with negative arousal, non-immediacy, tension, and anxiousness (Burgoon and Koper, 1984; Mann et al., 2020). Under circumstances that are moderately anxiety-provoking, the reticent individual may exhibit stress-related indicators such as increased fidgeting, adaptor gestures, elevated pitch and strident voice quality; under circumstances that are more anxiety-provoking, such as an interrogation, the communicator may go into “lock-down,” exhibiting the rigidity pattern associated with tension—flat affect, reduced facial and head expressivity, little vocal variety and the like.

Given the need to suppress information during deception, deception researchers have investigated behavioral cues associated with reticent communication. People often assume that nervousness is a sign of deceptiveness even if objectively, that is untrue (Vrij and Fisher, 2020). Although the proposition that deception can be revealed through shifty eyes has all but been disproven, cues associated with deception do overlap with ones associated with anxiety and nervousness. In particular, rigidity is a potential indicator of deception (Twyman et al., 2014; Pentland et al., 2017). Although this research does not necessarily align rigidity with nervousness, nervous behaviors are associated with rigidity, including kinesic cues (Gregersen, 2005) and vocal tension (Laukka et al., 2008). This is partly because deception is thought to increase cognitive load, and higher cognitive load reduces overall activation (Vrij and Fisher, 2020). However, previous studies showed mixed results in the associations between vocal variations and nervousness. Whereas Laukka et al. (2008) found no variation differences when specifically analyzing nervousness, Hagenaars and van Minnen (2005) found lower variation in pitch during episodes of fear versus happiness. Nervousness is potentially a more salient trait than composure to perceive in a group setting. In this context, nervousness is viewed as the bipolar opposite of composure. Given that the behaviors of a calm and collected group member may go unnoticed, participants in the current study were asked to rate the nervousness of group members. We hypothesize that perceptions of nervousness (either caused by social pressures or attempts to conceal information) will be exhibited in more rigid and tense behaviors.

In sum, we expect to replicate Burgoon and Le Poire (1999) and Pentland et al. (2017) in finding nonverbal indicators of nervousness and tension, as compared to composure, that include:

• Rigid and tense behaviors such as reduced head and face movements,
• Less immediacy (less gaze and indirect facing),
• Less vocal and kinesic pleasantness (e.g., less relaxed laughter and vocal resonance),
• Softer vocal amplitude, less vocal fluency,
• Higher pitch,
• Fewer and shorter turns-at-talk.

Left as a research question are the other vocal variations perceived as nervousness. Table 2: Hypothesized relationships between perceived nervousness and nonverbal behaviors, operationalizes the relationship between these principles and hypothesized nonverbal signals.

**TABLE 2:** Hypothesized relationships between perceived nervousness and nonverbal behaviors.

Principles	Strategies	Hypothesized nonverbal signals of nervousness
Withdrawn from engagement	Low expressivity	• Softer amplitude • More rigidity in facial animation • More rigidity in head movements
	Low conversational management	• Shorter talk time • Fewer turns-at-talk
Unpleasantness	Negativity	• Fewer pleasant facial expressions • Less nodding
	Non-immediacy	• Direct facing (not measurable in a group setting)
	Tension	• Higher pitch • Less vocal variation • Less vocal fluency • More rigidity in facial animation • More rigidity in head movements • Fidgety with hands and feet (not measured)

### Trust

Scholars from a variety of fields have studied trust through differing disciplinary lenses. Psychologists, for example, might emphasize the attributes of the individual that foster perceptions held by another, whereas sociologists might examine the relationships present within groups that lead to trust (Rousseau et al., 1998). At its core, trust is an *expectancy* about future behavior since one must assume that a person, group, or organization will behave in a particular way. If we trust a person, we are taking a risk and making ourselves vulnerable to them, so we want some assurance about what will happen when we do. Gottman (2011) argues this comes down to two factors: transparency and positive moral certainty. Transparency is the opposite of deceptiveness and allows us to rely on the other person when necessary. Positive moral certainty means we believe our partner is an ethical, moral person and will “treat us and others with high moral standards, integrity, honesty, kindness, love, and goodwill” (Gottman, 2011, p. 177). DeSteno (2014) argues that trust has two facets: integrity and competence. We are willing to trust someone who has both a high moral character and the expertise we need.

Trust always entails some level of risk, uncertainty, or willingness to be vulnerable through reliance and disclosure (van der Werff and Buckley, 2017). Simpson’s (2007) dyadic model of trust determines whether or not trust will result from an interaction between two interdependent actors or groups. The participants must be willing to take a risk and make themselves vulnerable for the sake of a mutually beneficial outcome. Each partner makes an independent assessment of whether the other is making decisions contrary to their own self-interest in favor of the best interests of the partner or the relationship (called “transformation of motivation”). Burgoon et al. (in press) recently posited an integrated adaptative “spiral model of trust.” The spiral model suggests that positive violations of expectancies (ones that conform to or surpass expectations) are more welcome than confirmations and therefore are more likely to foster trust than negative violations (ones that fail to meet expectations). These expectancy violations can take both verbal and nonverbal forms but our focus here is on the nonverbal ones. Boone and Buck (2003) proposed that emotional expressivity, the degree to which individuals accurately communicate their feeling states, helps to establish trustworthiness. Although existing research has found correlations between perceived trustworthiness and both verbal and nonverbal behavior (Wood, 2006; Lucas et al., 2016; Chen et al., 2020), people place overreliance on facial expressions when assessing credibility (Tsankova et al., 2012; Lucas et al., 2016).

What does trust look like, non-verbally? It can be expressed dyadically in the form of reciprocity, convergence, synchrony, or involvement that two partners share, or it can be examined individually in terms of the amount of uncertainty, tension, and suspicion that is expressed. Burgoon (in press) proposed that suspicion’s relationship to trust is curvilinear in that it is associated with the highest degree of uncertainty. As uncertainty is reduced, suspicion either morphs into distrust (greater certainty about the other’s untrustworthiness) or trust (greater certainty about the other’s trustworthiness) (Toma and Hancock, 2012). Once the nature of another person’s motives become known, whether or not they are trustworthy becomes known. Furthermore, we expect trust to correlate with dominance positively, because dominant individuals tend to convey confidence and appear competent (Anderson and Kilduff, 2009), and competence instills trust. Additionally, trust is expected to be associated with less nervousness, because individuals may employ nervousness as a heuristic when judging veracity, although nervousness may not imply lying (Feeley and deTurck, 1995; Vrij and Fisher, 2020). Consequently, we can hypothesize:

• Nonverbal indicators of dominance are positively associated with trust.
• Nonverbal indicators of tension and nervousness are negatively associated with trust.
• Uncertainty is negatively associated with trust.
• Nonverbal indicators of involvement and immediacy are positively associated with trust, specifically,

(1) High amounts of gaze, (2) direct facing, (3) forward lean, (4) rapid speech, (5) short response latencies, (6) fluent speech, and (7) long turns-at-talk.

## Materials and Methods

### Participants

We conducted 95 experimental sessions at eight universities around the world. Due to video recording failures, we used 56 games and 379 players (166 males and 213 females) for this study. Specifically, this sample includes 9 games (61 players) from the Western United States, 6 games (41 players) from the Southwestern United States, 6 games (42 players) from the Northwestern United States, 3 games (20 players) from Israel, 8 games (58 players) from Fiji, 4 games (21 players) from Zambia, 10 games (66 players) from Singapore, and 10 games (70 players) from Hong Kong, China. Furthermore, participants recruited at the same site were culturally diverse because of recruitment of college students with international experiences and various tribal backgrounds. Age averaged 21.90 years (*sd* = 3.46 years), with 12 participants not reporting their age. Among the 366 participants who reported their ethnicities, 48.1% were Asian, 18.9% were white, 13.7% were Fijian, 6.6% were black, while Latin/Hispanic, multiracial and other individuals accounted for 5.5, 4.4, and 3%, respectively. Additionally, among the 368 participants who reported their native languages, 44.3% were native English speakers.

### Experiment Procedures

The experiment consisted of group interactions using a scenario modified from popular board games, Mafia (designed by Dimitry Davidoff) and the Resistance (designed by Don Eskridge). Groups of six to eight participants were seated equidistant from one another in a circle at desks, each with a laptop computer. Participants first took turns to introduce themselves and answer a follow-up question from another participant as an icebreaker activity. Next, two to three of them were randomly assigned the role of a spy, while the rest of the group were assigned the role of villagers. Villagers were to conduct missions to eliminate spies, who were attempting to infiltrate the village. Spies were to try to sabotage the missions. Villagers would win a point for each mission that succeeded; spies would win a point for each mission that failed. At various junctures, the players rated one another on the relational dimensions of dominance, nervousness, and trustworthiness. Because the spies were working against the interest of villagers, variance was introduced in how players judged one another. Only the spies knew who the other spies were, creating uncertainty among villagers as to who to trust. In this sample, 229 players were assigned to be villagers, and 150 were spies; 53.6% of them had played a game similar to our experiment scenario before. Villagers won 28 games.

The game consisted of up to eight rounds and was capped at 1 h. For each round, participants were asked to complete missions in a hypothetical town. First, they elected a leader. The leader then chose a team of three to five (depending on the size of the group and how many rounds had been played) that had to be approved by a majority vote from the group. Next, teams “completed” the mission through anonymous votes. Villagers were always expected to vote for the missions to succeed. Spies were expected to vote to fail the missions, although they might vote strategically for a mission to succeed. One or two failed votes caused the mission to fail, depending on the size of the group and how many rounds had been played. The final winners were those who won more rounds, which earned team members monetary rewards. In addition, elected leaders would earn extra money. Audio-visual signals from each player were recorded during the entire game, including the icebreaker activity (see Dorn et al., in press, for a complete description of the experimental protocol).

### Independent Variables – Nonverbal Behaviors

The nonverbal behavioral features covered in this study include facial, head pose, and vocalic features. To extract facial features, videos were captured from front-facing cameras built into the computer tablets, an overhead 360-degree camera, and a webcam that recorded the entire group interaction. We fed the video recordings of every player into OpenFace, an open-source deep-neural-network (DNN) based facial recognition tool (Baltrusaitis et al., 2018), which output an intensity score (from zero to five) of 17 facial action units (FAUs) for each frame. We calculated the mean and standard deviation of these 17 FAUs for every player and game phase. Table 3: Facial action units (AU) output by OpenFace lists the names of the FAUs (Ekman and Friesen, 1978). OpenFace also output three features that represent the 3D location of the head with respect to camera and three features of head rotation (i.e., pitch, yaw, and roll). The time to take within-game surveys was excised from each video. OpenFace developers conducted extensive experiments to demonstrate the tool’s state-of-the-art performances on FAU detection and head pose estimation (Baltrusaitis et al., 2018), thus our analysis results are reliable to the extent that OpenFace is a valid tool.

**TABLE 3:** Facial action units (AU) output by OpenFace.

AU number	Description
1	Inner brow raiser
2	Outer brow raiser
4	Brow lowerer
5	Upper lid raiser
6	Cheek raiser
7	Lid tightener
9	Nose wrinkler
10	Upper lip raiser
12	Lip corner puller
14	Dimpler
15	Lip corner depressor
17	Chin raiser
20	Lip stretcher
23	Lip tightener
25	Lips part
26	Jaw drop
45	Blink

**FIGURE 1:**
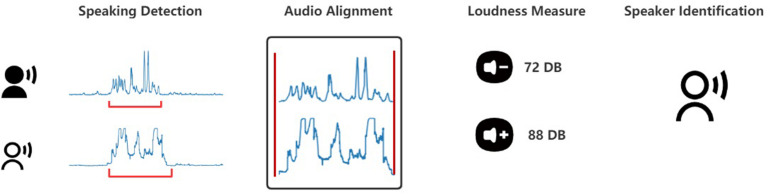
Pipeline for segmenting audio files of players’ turns-at-talk.

To extract vocalic features, we first developed a pipeline, a procedure for speech detection and audio alignment to segment audio files of players’ turns-at-talk. Specifically, we started with detecting speech in audio recordings of each player. Because audio waves picked up by each microphone were slightly different due to different distances between microphones and speakers, we employed an audio alignment algorithm named dynamic time warping to align the audio waves of the same utterance. Then, we identified speakers based on the highest loudness, because the loudest recording should be picked by the microphone assigned to the speaker. Finally, we segmented audio files into players’ turns-at-talk. Figure 1 summarizes the pipeline.

The audio segments were processed by OpenSmile, a software tool for automatic feature extraction from audio signals (Eyben and Schuller, 2015). Based on the demonstrations of the validity of OpenSmile for multimedia recognition tasks by Eyben et al. (2013), we judged OpenSmile to be a valid tool for our analysis.

Table 4: Acoustic measures and descriptions lists the turn-at-talk vocalic features output from OpenSmile used in our multilevel regression analyses. These features were averaged for each game phase. Additionally, we standardized speaking tempo by dividing the word count by speech time for each player in every game phase. Because of the high costs associated with obtaining accurate transcripts, we used a subset of 28 games whose transcripts were available for further analysis of speaking tempo. Lastly, we standardized all the nonverbal behavioral features within the same game session and the same game phase.

**TABLE 4:** Acoustic measures and descriptions.

Measure name	Description
F_0_ (pitch) Mean F_0_ (pitch) Std	The low to high level of a tone perceived by humans as pitch.
Loudness-Mean Loudness-Std	The amplitude of sound pressure perceived as loudness.
Turn-at-talk Duration	Duration of a turn-at-talk in seconds

### Dependent Variables – Ratings

To measure the relational dimensions, participants rated each other on dominance, composure, and trustworthiness on five-point scales. The survey items are described below. The questions incorporated several of the typical bipolar adjectives used to measure each dimension. To avoid fatigue, these were combined into single item measures.

• Please rate how dominant each player was during this round. Were they active and forceful or passive and quiet? A rating of 5 would mean you thought they were assertive, active, talkative, and persuasive. A score of 1 would mean you thought they were unassertive, passive, quiet and not influential. Please mark any number from 1 to 5.
• Please rate how nervous each player was during this round. A rating of 5 would mean you thought they were anxious, uncomposed, and tense. A rating of 1 would mean you thought they were calm, composed, and relaxed. Please mark any number from 1 to 5.
• Please rate how much you trusted each player during this round. Were they trustworthy or suspicious? A rating of 5 would mean they were honest, reliable and truthful and 1 would mean you thought they were dishonest, unreliable and deceitful.

Ratings were collected after the icebreaker and every two rounds. This allowed measurement of dynamics in the relational messages. Because games varied in the number of rounds, all games were segmented into three game phases, namely, the icebreaker (phase 1), Round 1 and 2 (phase 2) combined, and Round 3 till the end of the game, combined (phase 3). For each game phase, villagers’ ratings of each player were averaged separately on dominance, nervousness, and trustworthiness. Ratings from spies were excluded because of contamination by their knowledge of others’ game roles. Additionally, self-ratings were excluded.

## Results

### The Mixed-Effects Regression Model

To test the relationships among dominance, nervousness, trust and nonverbal behaviors, multivariate mixed-effects regression models were specified for each of the dependent variables of dominance, nervousness and trust perceptions. Control variables included game phase (phase 1, phase 2, or phase 3), game role (spy or villager), gender (male or female), previous game experience (yes or no), English as a second language (yes or no), and game score difference between spies and villagers by game phase. The interaction effect between game phase and game role was included because perceptions of players with different roles may have had different trends as the games progressed. As shown in the Supplementary Material, spies were perceived as less dominant (Supplementary Tables 1–3) and less trustworthy (Supplementary Tables 7–9) over time. Individual nonverbal behaviors were set as independent variables and their unique game name was specified as a random effect. Equation 1 specifies the regression equation.

Equation 1: Mixed-Effects Regression Model

R⁢e⁢l⁢a⁢t⁢i⁢o⁢n⁢a⁢l⁢M⁢e⁢s⁢s⁢a⁢g⁢e⁢S⁢c⁢o⁢r⁢e=G⁢a⁢m⁢e⁢P⁢h⁢a⁢s⁢e+G⁢a⁢m⁢e⁢R⁢o⁢l⁢e+G⁢a⁢m⁢e⁢P⁢h⁢a⁢s⁢e×G⁢a⁢m⁢e⁢R⁢o⁢l⁢e+G⁢e⁢n⁢d⁢e⁢r+G⁢a⁢m⁢e⁢E⁢x⁢p⁢e⁢r⁢i⁢e⁢n⁢c⁢e+N⁢a⁢t⁢i⁢v⁢e⁢L⁢a⁢n⁢g⁢u⁢a⁢g⁢e

  +G⁢a⁢m⁢e⁢S⁢t⁢a⁢t⁢u⁢s+N⁢o⁢n⁢v⁢e⁢r⁢b⁢a⁢l⁢B⁢e⁢h⁢a⁢v⁢i⁢o⁢r+(1|G⁢a⁢m⁢e)+ϵ

Table 1: Hypothesized relationships between perceived dominance and nonverbal behaviors and Table 2: Hypothesized relationships between perceived nervousness and nonverbal behaviors represent a theoretical delineation of dominance and nervousness principles and hypothesized nonverbal signals. Trust signals are presumed to draw from the dominance and nervousness indicators. The tables present an expansive look at nonverbal signals available from full frontal videos from the shoulders up. Due to data collection constraints or behavioral coding limitations, some behaviors are not included in the current data analysis. Specifically, features related to eye behavior, trunk and limb movement, and interactional dynamics (non-reciprocation and self-nomination) are excluded from the analysis.

Table 5: Mixed-effect regression results for nonverbal behaviors related to dominance presents test results for each nonverbal behavior analyzed with respect to perceptions of dominance. For simplicity, the results of control variables are omitted.

**TABLE 5:** Mixed-effect regression results for nonverbal behaviors related to dominance.

Principle	Strategies	Hypothesized	Measurement	β(*SE*)
Physical potency	Size of strength	Louder voice	Mean vocal loudness	0.157 (0.03)***
		Deep-pitched voice	Mean vocal pitch	−0.04 (0.03)
		Upright head and posture	Mean head pitch	−0.008 (0.03)
			Mean head yaw	−0.017 (0.03)
			Mean head roll	0.017 (0.03)
	Expressivity	More facial expression	SD AU01	0.054 (0.03)*
			SD AU02	0.064 (0.03)*
			SD AU04	0.006 (0.03)
			SD AU05	0.059 (0.03)*
			SD AU06	0.075 (0.03)**
			SD AU07	0.029 (0.03)
			SD AU09	0.067 (0.03)**
			SD AU10	0.072 (0.03)**
			SD AU12	0.043 (0.03)^+^
			SD AU14	0.123 (0.03)***
			SD AU15	0.106 (0.03)***
			SD AU17	0.057 (0.03)*
			SD AU20	0.059 (0.03)*
			SD AU23	0.108 (0.03)***
			SD AU25	0.126 (0.03)***
			SD AU26	0.085 (0.03)***
			SD AU45	0.075 (0.03)**
		More variation in pitch	SD vocal pitch	−0.047 (0.03)^+^
		More head movement	SD head pitch	0.12 (0.03)***
			SD head yaw	0.069 (0.03)**
			SD head roll	0.074 (0.03)**
		More rapid speaking tempo	Word count/speaking time	0.029 (0.039)
Resource control	Possession of valued commodities	More turns-at-talk	Count of turn-at-talk	0.34 (0.024)***
		Longer turns at talk	Mean turn-at-talk duration	0.153 (0.025)***

^+^p < 0.1, *p < 0.05, **p < 0.01, ***p < 0.001. Only beta weights and standard errors for each nonverbal behavior are presented. See Supplementary Tables 1–3 for full model results.

Results indicate that perceptions of dominance are associated with a louder voice, more expressive facial behavior, more head movement, and more and longer turns-at-talk. Vocal pitch, head position, and speaking tempo were not found to be significantly related to perceptions of dominance.

Next, we tested relationships between selected nonverbal behaviors and nervousness. Table 6: Mixed-effect regression results for nonverbal behaviors related to nervousness presents these results. Generally, we expect to see withdrawn and tense behaviors.

**TABLE 6:** Mixed-effect regression results for nonverbal behaviors related to nervousness.

Principle	Strategies	Hypothesized	Measurement	β(*SE*)
Withdrawn from engagement	Low expressivity	Softer amplitude	Mean vocal loudness	−0.038 (0.02)^+^
		More rigidity in facial animation	SD AU01	−0.013 (0.02)
			SD AU02	−0.06 (0.02)**
			SD AU04	0.033 (0.02)
			SD AU05	−0.013 (0.02)
			SD AU06	−0.006 (0.02)
			SD AU07	−0.014 (0.02)
			SD AU09	0.002 (0.02)
			SD AU10	−0.022 (0.02)
			SD AU12	0 (0.02)
			SD AU14	−0.032 (0.02)
			SD AU15	−0.038 (0.02)
			SD AU17	−0.005 (0.02)
			SD AU20	−0.024 (0.02)
			SD AU23	−0.031 (0.02)
			SD AU25	−0.032 (0.02)
			SD AU26	−0.037 (0.02)
			SD AU45	−0.031 (0.02)
		More rigidity in head movement	SD head pitch	−0.05 (0.02)*
			SD head yaw	−0.044 (0.02)^+^
			SD head roll	−0.061 (0.02)**
	Low conversational management	Small amount of talk time	Mean turn-at-talk duration	−0.052 (0.02)*
		Fewer turns-at-talk	Count of turn-at-talk	−0.063 (0.02)**
Unpleasantness	Tension	Higher pitch	Mean vocal pitch	0.038 (0.03)
		Less vocal variation	SD vocal pitch	0.032 (0.02)

^+^p < 0.1, *p < 0.05, **p < 0.01. *Only beta weights and standard errors for each nonverbal behavior are presented. See Supplementary Tables 4–6 for full model results.*

The results show that nervousness is associated with more rigid head movements, and fewer and shorter turns-at-talk. Measures associated with loudness, pitch, vocal variation, and facial animation were not found to be significant.

Lastly, the correlation between trust and dominance is 0.31 (*p* < 0.001), and the correlation between trust and nervousness is −0.082 (*p* < 0.01). Both correlations are in the same direction as expected, but the relationship between trust and nervousness accounts for virtually no variance, indicating these measures are independent. Table 7: Mixed-effect regression results for nonverbal behaviors related to trust presents the relationship between trust and the hypothesized nonverbal behaviors. We expect to see indicators of trust similar to those of dominance and opposite to those of nervousness.

**TABLE 7:** Mixed-effect regression results for nonverbal behaviors related to trust.

Dominance indicators	Nervousness indicators	Hypothesized for trust	Measurement	β(*SE*)
Louder voice	Softer amplitude	Louder voice	Mean vocal loudness	0.026 (0.02)
Deep-pitched voice	Higher pitch	Deep-pitched voice	Mean vocal pitch	−0.011 (0.03)
Upright head and posture		Upright head and posture	Mean head pitch	−0.021 (0.02)
			Mean head yaw	−0.010 (0.02)
			Mean head roll	−0.020 (0.02)
More facial expression	More rigidity in facial animation	More facial expression (i.e., less rigidity in facial animation)	SD AU01	−0.026 (0.02)
			SD AU02	−0.010 (0.02)
			SD AU04	0.003 (0.02)
			SD AU05	−0.010 (0.02)
			SD AU06	−0.003 (0.02)
			SD AU07	−0.007 (0.02)
			SD AU09	0.004(0.02)
			SD AU10	0.041 (0.02)^+^
			SD AU12	0.003 (0.03)
			SD AU14	0.008 (0.02)
			SD AU15	0.004 (0.03)
			SD AU17	−0.022 (0.02)
			SD AU20	0.011 (0.02)
			SD AU23	−0.014(0.02)
			SD AU25	0.028 (0.02)
			SD AU26	0.020 (0.02)
			SD AU45	−0.039 (0.02)
More variation in pitch	Less vocal variation	More variation in pitch	SD vocal pitch	−0.002 (0.03)
More head movement	More rigidity in head movement	More head movement (i.e., less rigidity in head movement)	SD head pitch	−0.016 (0.03)
			SD head yaw	0.013 (0.02)
			SD head roll	0.000 (0.02)
More rapid speaking tempo		More rapid speaking tempo	Word count/speaking time	−0.090 (0.04)*
More turns-at-talk	Fewer turns-at-talk	More turns-at-talk	Count of turn-at-talk	0.051 (0.03)*
Longer turns at talk	Small amount of talk time	Longer turns at talk	Mean Turn-at-talk duration	0.054 (0.025)*

^+^p < 0.1, *p < 0.05. *Only beta weights and standard errors for each nonverbal behavioral are presented. See Supplementary Tables 7–9 for full model results.*

Results show that more, longer, and slower turns-at-talk are positively associated with perceptions of trust, while loudness, pitch, upright head and posture, and less rigidity in facial expressions and head movement did not correlate with perceptions of trust.

### Exploratory Analysis

Computational extraction of behavioral features provides insight into nonverbal behaviors that manual coding cannot. The current video corpus was processed with automated voice and face behavioral analysis software which produced 75 features. After calculating the mean and standard deviation of these features, our dataset resulted in 150 nonverbal behavior features. Although conducting separate statistical tests on this many features increases the probability of Type 1 errors, we do so with the intention of exploring macro-level patterns, not hypothesis testing. Table 8: Proportion of significant nonverbal features, Means and Table 9: Proportion of significant nonverbal features, Standard Deviations below provide insights into the relationships among dominance, nervousness, trust, and each category of features that had *p*-values below 0.05. The values in the table provide a count of the number of statistically significant features by dependent variable (dominance, nervousness, and trust), channel (face, head, or voice) and summary statistic (mean or standard deviation). The tables also count the number of positive or negative coefficients.

**TABLE 8:** Proportion of significant nonverbal features, means.

	Summary statistic = Mean		
	# Features *p* < 0.05/# Features	# Positive Coef./# Features *p* < 0.05	# Negative Coef./# Features *p* < 0.05
**Dominance**	**31/75**	**17/31**	**14/31**
Face	12/17	12/12	0/12
Head	1/6	0/1	1/1
Voice	18/52	5/18	13/18
**Nervousness**	**6/75**	**4/6**	**2/6**
Face	3/17	1/3	2/3
Head	0/6	0	0
Voice	3/52	3/3	0/3
**Trust**	**2/75**	**1/2**	**1/2**
Face	0/17	0	0
Head	0/6	0	0
Voice	2/52	1/2	1/2

**The positive (negative) coefficients indicate that a higher mean of the nonverbal feature is associated with a higher (lower) level of perception of dominance, nervousness, or trustworthiness*.*

**TABLE 9:** Proportion of significant nonverbal features, standard deviations.

	Summary statistic = Standard deviation		
	# Features *p* < 0.05/# Features	# Positive Coef./# Features *p* < 0.05	# Negative Coef./# Features *p* < 0.05
**Dominance**	**70/75**	**68/70**	**2/70**
Face	14/17	14/14	0/14
Head	6/6	6/6	0/6
Voice	50/52	48/50	2/50
**Nervousness**	**21/75**	**0/21**	**21/21**
Face	1/17	0/1	1/1
Head	2/6	0/2	2/2
Voice	18/52	0/18	18/18
**Trust**	**13/75**	**13/13**	**0/13**
Face	0/17	0	0
Head	0/6	0	0
Voice	13/52	13/13	0/13

**The positive coefficients indicate that a higher value for the standard deviation of the nonverbal feature (i.e., a higher degree of variability) is associated with a higher level of perceived dominance, nervousness, or trustworthiness. The negative coefficients indicate that the variable has a negative sign in the final equation such that less variability is associated with a higher degree of perceived dominance, nervousness or trustworthiness*.*

Dominance was associated with the greatest number of significant features with 101. Nervousness had far fewer with 27 significant features, and trust, with only 15 significant features. Among the 143 significant relationships, 104 were standard deviations and 39 were means, which indicates that the variation, rather than the average level of nonverbal signals, influenced the perceptions of relational dimensions more.

Interestingly, when looking at the count of positive or negative coefficients associated with measured standard deviation (Table 9: Proportion of significant nonverbal features, Standard Deviations), we see far more significant features with positive coefficients for dominance and almost all with negative coefficients for nervousness. In the case of standard deviations, positive coefficients correspond to more dynamic behaviors and negative coefficients correspond to muted or rigid behaviors. Almost all (68/70) of the significant standard deviation measures were positive for dominance, and all (21/21) were negative for nervousness. Again, although we expect that some Type 1 errors are likely, this finding supports our general hypothesis that dominance is associated with more energetic behaviors (more variability) and nervousness is associated with tension (less variability). Furthermore, a majority (13/15) of significant features for trust were standard deviations of voice features, and they all had a positive coefficient, indicating that trust tends to be associated with more variability in voice.

### Classification Results

Our analysis revealed the significant effects of the nonverbal signals on relational dimensions. However, statistical significance does not necessarily imply practical significance. In this section, we aim to predict the relational scores with behavioral measures. Given the reported significance of many nonverbal signals, we assume that such variables will help to predict the relational dimensions.

To formulate the prediction of relation dimensions as a classification problem and to mitigate individual level rating bias, we binarized the aggregated score of the three relational dimensions. The median of each player’s response was used as the cutoff to dichotomize the original scores. In this way, labels of “High/Low dominance,” “High/Low nervousness,” and “High/Low trustworthiness” were assigned to each player, and the generated categories were roughly balanced.

The same nonverbal variables in the previous analysis were used as predictors. Six popular machine learning algorithms, Logistic Regression, Random Forest, Naïve Bayes, Support Vector Machine (SVM), and two ensemble learning methods, Bagging and Boosting, were used to predict the binary categories of dominance, nervousness and trustworthiness. Ensemble methods, which combine multiple learning algorithms to achieve better prediction, have gained wide popularity due to their superior performance. The two ensemble methods that we used combine multiple decision trees to make the final decision. An 80/20 split was applied to construct the training set from which the model was then applied to the test set. To obtain a more reliable estimate of the model’s prediction ability, we adopted a “repetitive random split” strategy, that is, we randomly split the full data set 100 times. For each train-test split, the accuracy and F1 score of this model was recorded. The F1 score averages accuracy in predicting dominance and non-dominance, nervousness and composure, or trust and distrust. The mean of accuracy and F1 score over 100 splits was used to evaluate the model’s prediction performance. A modified stepwise variable selection (MSVS) method was used to search through the gigantic model space (Draper and Smith, 1981).

Table 10: Prediction accuracy and F1score of the machine learning models (RF, Random Forest; LR, Logistic Regression; NB, Naïve Bayes; BAG, Bagging; XGB, Boosting) summarizes the best accuracy and F1 score that our models achieved in the three prediction tasks. In our results, the highest accuracies on predicting dominance, nervousness and trustworthiness were all achieved by the bagging models, which shows its superiority as an ensemble method. The naïve Bayes models were outperformed by the bagging models only by a narrow margin, and their F1 scores were even higher when predicting dominance and nervousness. One consistent observation across different machine learning algorithms is that higher accuracies and F1 scores were attained when predicting dominance than when predicting nervousness and trustworthiness, which implies that perceived dominance is the most predictable relation dimension with nonverbal signals. On the other hand, the manifestations of nervousness and trustworthiness in nonverbal behaviors were more subtle and dynamic and cannot be accurately reflected in our aggregated predictors.

**TABLE 10:** Prediction accuracy and F1 score of the machine learning models (RF, random forest; LR, logistic regression; NB, Naïve Bayes; BAG, bagging; XGB, boosting).

Relational dimension	RF F1	RFACC	LR F1	LR ACC	SVM F1	SVM ACC	NB F1	NB ACC	BAG F1	BAG ACC	XGB F1	XGB ACC
Dominance	0.61	0.70	0.63	0.72	0.63	0.72	0.69	0.72	0.69	0.72	0.68	0.70
Nervousness	0.44	0.62	0.46	0.66	0.45	0.66	0.58	0.66	0.56	**0.66**	0.58	0.65
Trustworthiness	0.43	0.63	0.43	0.66	0.43	0.66	0.59	0.65	0.59	**0.66**	0.58	0.65

*The accuracies of the XGB (bagging) model (in bold) were highest among the six machine learning classifiers.*

**FIGURE 2:**
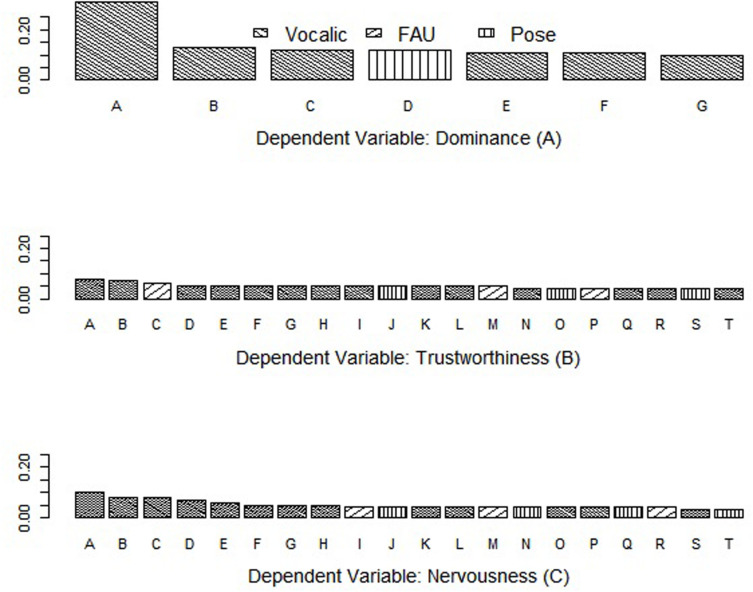
Variable importance reported by the best random forest model on predicting Dominance **(A)**, Trustworthiness **(B)**, and Nervousness **(C)**.

Since variable importance can be output by some decision tree-based models, we further examined which variables mattered most to the prediction of relational dimensions by calculating the means of variable importance reported by the best random forest models. The results are presented in Figure 2. The best random forest model of predicting dominance contains 7 nonverbal signals, and 6 of them are vocalic variables. The leftmost bar in Figure 2A represents the most prominent predictor, which is the summation of turns-at-talk of a player. The variable importance analyses of trustworthiness prediction (Figure 2B) and nervousness prediction (Figure 2C) exhibit a similar pattern: the majority of the most important variables are vocalic features. Due to the models’ limited predictive power, the variable importance scores were all very low. However, the importance of vocalic signals in the impressions of relational dimensions can be inferred from these figures.

Table 11: Most important variables in the best Random Forest model reports the most important variables when predicting the perceived dominance, trustworthiness and nervousness, respectively. The rank was based on each variable’s importance given by the random forest models.

**TABLE 11:** Most important variables in the best random forest model.

Dependent variable	Importance rank	Variable name	Variable category	Variable description
**Dominance**	**A**	Turn-at-talk Duration Summation	Vocalic	The total duration of turns-at-talk for a player
	**B**	**MFCC Channel 1 Standard Deviation**	Vocalic	The standard deviation of the first spectral envelope of MFCC
	**C**	LSP Channel 5 Derivative Standard Deviation	Vocalic	The standard deviation of the derivative of fifth line spectral pair frequency
	**D**	AU15 Intensity Standard Deviation	FAU	The standard derivation of the intensity of the fifteenth facial action unit
	**E**	Fundamental Frequency Mean	Vocalic	The mean of fundamental frequency
	**F**	MFCC Channel 1 Derivative Standard Deviation	Vocalic	The standard deviation of the derivative of the first spectral envelope of MFCC
	**G**	LSP Channel 1 Mean	Vocalic	The mean of the first line spectral pair frequency
**Trustworthiness**	**A**	**Loudness Derivative Standard Deviation**	Vocalic	The standard deviation of the derivative of the normalized loudness
	**B**	ZCR Standard Deviation	Vocalic	The standard deviation of the zero-crossing rate of time signal
	**C**	AU 20 Intensity Mean	FAU	The mean of the intensity of the 20th facial action unit
	**D**	MFCC Channel 12 Derivative Standard Deviation	Vocalic	The standard deviation of the derivative of the 12th spectral envelope of MFCC
	**E**	**MFCC Channel 11 Derivative Standard Deviation**	Vocalic	The standard deviation of the derivative of the 11th spectral envelope of MFCC
	**F**	Fundamental Frequency Derivative Mean	Vocalic	The mean of the derivative of fundamental frequency
	**G**	Fundamental Frequency Derivative Standard Deviation	Vocalic	The standard deviation of the derivative of fundamental frequency
**Nervousness**	**A**	MFCC Channel 1 Standard Deviation	Vocalic	The standard deviation of the first spectral envelope of MFCC
	**B**	MFCC Channel 1 Derivative Mean	Vocalic	The mean of the derivative of the first spectral envelope of MFCC
	**C**	Loudness Derivative Standard Deviation	Vocalic	The standard deviation of the derivative of the normalized loudness
	**D**	LSP Channel 1 Derivative Standard Deviation	Vocalic	The standard deviation of the derivative of the first line spectral pair frequency
	**E**	MFCC Channel 11 Derivative Standard Deviation	Vocalic	The standard deviation of the derivative of the 11th spectral envelope of MFCC
	**F**	Voice Probability Derivative Standard Deviation	Vocalic	The standard deviation of the derivative of the voicing probability
	**G**	MFCC Channel 2 Derivative Standard Deviation	Vocalic	The standard deviation of the derivative of the 2nd spectral envelope of MFCC

**“sma” indicates that the variable is smoothed by a moving average filter with window length 3. (2) A bolded variable name indicates that this variable appeared twice in Table*.*

A few model-free observations can be drawn. First, the majority of the important predictive variables comes from the vocalic signals. The only two non-vocalic variables are the mean of AU 15 (lip corner depressor) and the standard variation of AU 20 (lip stretcher), both of which, interestingly, are lip movements. Secondly, when predicting trustworthiness and nervousness, most of the important variables are standard deviations of an original measurement, which is consistent with the findings of the linear models. A few variables (bold in Table 11: Most important variables in the best Random Forest model) appeared twice in Table 11, for example, MFCC Channel 1 Standard Deviation, which represents the standard deviation of the first spectral envelop of MFCC, and Loudness Derivative Standard Deviation, which reflects the standard deviation of vocal loudness. The co-occurrence of such variables not only demonstrates the robustness of our analysis, but also calls for efforts to interpret these nonverbal signals.

## Discussion

Relational communication is a fundamental aspect of interpersonal communication and nonverbal signals are a centerpiece of understanding that endeavor. How people regard one another and their relationship can be expressed in ways that speak what words cannot. Nonverbal relational messages express meanings and sentiments that people may refrain from saying out loud, such as declaring a romantic interest or expressing schadenfreude, or can ease the burden of delivering hurtful messages such as the end of a relationship, a death in the family or a terminal cancer diagnosis.

Relational communication can take myriad forms, as enumerated by Burgoon and Hale (1984, 1987; Hall et al., 2005). Only three are the focus here, three that coincide with our investigation of cross-cultural group deception, but it should be understood that the diverse themes of relational messages all entail nonverbal signals to varying degrees and so are subject to the same opportunities and obstacles that we discuss here.

A major impetus for featuring relational communication in this special issue is that the development of new automated tools for measuring nonverbal signals and new machine learning methods for analyzing them has made it possible to delve into the heretofore elusive and ephemeral topic of relational messaging. We often know when a significant message has been exchanged between two people, we just cannot always put our finger on what its basis is. The current investigation begins to put “flesh” on the skeleton of relational communication, to discover the possibilities of examining nonverbal communication in a more microscopic way than in the past and to discover what obstacles we encounter along the way. At the same time, this synergistic effort brings together computer science, psychology and communication methods in social signal processing. This direction is at the forefront of work on affective computing and neurocognitive psychology. The outgrowth of this program of research is that technical fields will benefit from theories originating from psychology and communication, and the social sciences will benefit from the technological advances of computer science fields. For example, predictive models using input features suggested by social sciences may facilitate understanding relational messages in real time and designing interactive systems that recognize and interpret human affects. Meanwhile, using machine-learning based automated tools for measurement is more scalable and less costly than manual coding, and these tools are helpful for testing and refining social science theories. Additional side benefits of the current project are that it situated nonverbal interaction in a group setting rather than the usual dyadic one and explored such interaction in natural, ongoing discussion rather than scripted or brief interchanges. This has brought with it the messiness that accompanies naturalistic human interaction and is the basis for many of the obstacles, and mitigating strategies, we will discuss.

### Nonverbal Signals of Dominance and Non-dominance

Of the three relational message themes examined in this paper, dominance was associated with the widest variety and greatest number of cues. It was associated with 101 of the 150 variables tested, whereas the other two relational themes, composure and trust, were associated with far fewer. The results indicate that perceptions of dominance were associated with a louder voice, more expressive facial behavior, more head movement (in terms of pitch, yaw, and roll), and more and longer turns-at-talk. Additionally, several of the FAUs were associated with dominance as well; of the 17 we measured, 15 of them were associated with perceptions of dominance. Dunbar (2004) argued that while power is a perception based on a relationship and the resources to which one has access, dominance is based in the particular contextual behaviors that one enacts during a relationship and interaction. That was certainly true in this case where the objectively measured behaviors that have been commonly associated with dominance in the research literature (see e.g., Dunbar and Burgoon, 2005) were related to the perceptions that one was behaving dominantly.

### Nonverbal Signals of Nervousness and Composure

Nonverbal signals of nervousness were associated with softer vocal amplitude, less head movement, and fewer and shorter turns-at-talk. The hypothesis that nervousness is reflected in rigid facial animation, higher pitch, and less vocal variation was not confirmed in the present study. However, overall results largely support that nervousness leads to tense and rigid nonverbal behaviors. The exploratory analysis showed that all significant face, head, and voice features were associated with reduced variation. Most notably, all 18 voice measures with a *p*-value below 0.05 had a negative coefficient, which indicates that perceptions of nervousness are amplified as vocal animation decreases. In the current study, the vocal channel seems to have been the predominant signal of nervousness. This is likely due to kinesic controllability and how emotional indicators in the voice are difficult to conceal. Additionally, markers of nervousness in the face and head are likely better assessed at critical moments during interactions such as immediately before or after turns-at-talk. Analysis over a wide timespan, such as the case in this study, may obscure subtle indicators of nervousness.

Surprisingly, vocal pitch was not a significant indicator of nervousness in our study. A possible explanation is that the manifestation of nervousness in voice may be confounded by one’s phonatory attributes, which correlate with gender, age, native language and even cultural background. Though these factors have been controlled in our model, the chance of nervousness’s effect being weakened by human’s highly varied vocal timbre still exists. It might be more meaningful to conduct within-subject vocalic analysis (e.g., compare utterances from the same individuals when they are nervous and those when they are not) to reveal the effect of nervousness. Another explanation is that nervousness would be more evident in dyadic interaction where an individual is the sole target of scrutiny and suspicion. In a group, it is easy to deflect attention to self by passively avoiding turns at talk or focusing attention on others, thus reducing one’s cognitive load and anxiety.

### Nonverbal Signals of Trust and Suspicion

Of the three relational message themes studied here, trust and its converse of distrust or suspicion, had the fewest nonverbal signals that predicted it. Only two single mean vocal features were associated with trust and 13 vocalic standard deviations were, all pointing to more variation in the voice contributing to the perception of trustworthiness. No facial or head features predicted trust, and none of the nonverbal features predicted distrust. This coincides with finding few features in the machine learning models as important for classifying high or low trust. Of the machine learning models, the bag-of-words methods achieved the highest, but paltry, 66% accuracy that was virtually the same as all the other methods. The F1 score was highest at 59%, indicating that averaging the accuracy scores for trust and distrust reduced overall accuracy. Except for those features of trust that overlapped with the significant predictors of dominance or composure, then, trustworthiness was clearly not easily predictable from nonverbal signals.

Why might that be? Is it the case that nonverbal features are not intrinsic ingredients in gauging who might be viewed as trusted? We do not think so. There are many possible explanations for the minimal appearance of nonverbal signals in the alchemy of trust. First is the fact that because participants had little basis for making judgments, group members tended to rate everyone similarly at the start. This would have created a restriction in range statistically. Secondly, because group members did not know one another, aggregating across time periods and moment-to-moment changes in behavior may have blurred any important signals into one average and meaningless soup. Were we to design a new study, we would seek to develop more nuanced measures of trust reflective of specific actions of what might have engendered trust or piqued suspicion, much like studies of close relationships seek to identify turning points or significant events in ongoing communication.

Third, and contrariwise, our moment-to-moment measurements were related to specific blocks of rounds, yet trust may grow out of the accretion of actions across time, something our measurement did not capture well. For example, by the end of the game, a villager could recall which time another player had been on a winning or failed mission and so make reasonable guesses about who were villagers and who, spies. This historical information had nothing to do with that player’s nonverbal actions at that point in time. This possibility points to the importance of selecting time slices that best reflect the granularity of the question of interest and deciding whether measurements should be geared to microscopic moments or a broader sweep of time. They are also a reminder that our understandings are best achieved by combining verbal and nonverbal information as well as other contextual information in our models.

Measurement error and the relatively simple variable construction may also account for the biases in our modeling results. The measurement error may come from multiple sources. If the subject’s face was not captured by the camera (for example, due to large amplitude of body movement) or multiple faces appeared in the same frame, the OpenFace software would output invalid measurements. Tablets can be slightly moved by a pressing finger, which results in drastic fluctuations in the head pose measurements. The performance of the SOTA audio diarization algorithm was also far from being perfect when being applied to our data set. As a result, the audio files generated in our pipeline might not reflect the subject-level utterances exactly. Considering such measurement errors, the effect size and significance level of some nonverbal signals may not reflect the reality. On the other hand, the bi-round-level aggregated mean and standard deviation may not represent the subtlety of nonverbal signals well either. Intuitively, the nonverbal messages matter most to the receivers’ perception when the sender is having the group’s attention. As a result, nonverbal analysis focusing on certain “critical moments” may be more meaningful, and this can be accomplished by narrowing analysis to specific segments of interaction.

Although automated tools could be employed to measure nonverbal behaviors, manual methods were still necessary for obtaining a few features accurately given the current technologies. For example, we required an accurate and precise count of words to calculate speaking tempo. Machine-generated transcripts were of low quality because of crosstalk, background noise, and accented speech, so we resorted to tedious manual correction. Furthermore, hesitations and interruptions could be identified easily by human coders, but those marked by automated transcription services did not fully correspond to what human coders would perceive as disfluencies. Because of the large amount of data, we did not manually code hesitations and interruptions for our analysis. However, these are important nonverbal features that could affect perceptions of dominance, nervousness, and trustworthiness. Future research could profitably explore reliable automated tools for measuring these nonverbal features.

## Summary

In summary, nonverbal signals color the meanings of interpersonal relationships. Humans rely on facial, head, postural and vocal signals to express relational messages of dominance or non-dominance. They rely on vocal signals to convey nervousness or composure. And to a lesser extent, some of these signals contribute to meanings of trust and distrust. Emerging automated analysis tools and machine learning methods have made possible a deeper understanding of the dynamics of relational communication and have exposed much of the messiness of studying communication under naturalistic conditions. Improvements in measurement and experimental design may mitigate some of these complications in the future.

## Data Availability Statement

The datasets generated for this study are not readily available because privacy restrictions exist and data is not available until the end of grant. Requests to access the datasets should be directed to JB, judee@arizona.edu.

## Ethics Statement

The studies involving human participants were reviewed and approved by the Institutional Review Board (IRB) at UC Santa Barbara. The patients/participants provided their written informed consent to participate in this study.

## Author Contributions

All authors listed have made a substantial, direct and intellectual contribution to the work, and approved it for publication. All authors have contributed equally to this work.

## Conflict of Interest

JB is affiliated with Discern Science International, a for-profit entity that develops systems for credibility assessment. The remaining authors declare that the research was conducted in the absence of any commercial or financial relationships that could be construed as a potential conflict of interest.
