# Supplementary material for: Nonverbal Behaviors “Speak” Relational Messages of Dominance, Trust, and Composure
Source: Front Psychol. 2021 Jan 26;12:624177. doi: 10.3389/fpsyg.2021.624177 (PMC7870468; doi:10.3389/fpsyg.2021.624177)
Supplement: Supplementary file 1 [file Table_1.pdf]

Supplementary Table 1: Mixed-effect regression results for nonverbal behaviors related to dominance (Model 1-10)

	Dependent variable:									
	Dominance									
	1	2	3	4	5	6	7	8	9	10
T2 (Round 1 and 2)	-0.028	-0.040	-0.039	-0.039	-0.039	-0.038	-0.039	-0.040	-0.041	-0.041
	(0.073)	(0.074)	(0.074)	(0.074)	(0.074)	(0.074)	(0.074)	(0.074)	(0.074)	(0.074)
T3 (After Round 2)	0.045	0.053	0.051	0.051	0.052	0.051	0.052	0.051	0.050	0.045
	(0.071)	(0.073)	(0.073)	(0.073)	(0.073)	(0.073)	(0.073)	(0.073)	(0.073)	(0.073)
Game Role (Spy = 1)	0.055	0.058	0.058	0.058	0.061	0.061	0.061	0.059	0.059	0.057
	(0.078)	(0.079)	(0.079)	(0.079)	(0.080)	(0.079)	(0.079)	(0.079)	(0.079)	(0.079)
Gender (Male = 1)	0.268***	0.238***	0.280***	0.276***	0.277***	0.265***	0.259***	0.279***	0.284***	0.301***
	(0.050)	(0.059)	(0.052)	(0.051)	(0.051)	(0.051)	(0.051)	(0.051)	(0.051)	(0.051)
Game Experience	0.081	0.067	0.071	0.069	0.070	0.076	0.081	0.071	0.082	0.070
	(0.056)	(0.057)	(0.057)	(0.057)	(0.057)	(0.057)	(0.057)	(0.057)	(0.057)	(0.057)
Native English Speaker	0.145*	0.176**	0.179**	0.179**	0.179**	0.175**	0.176**	0.177**	0.182**	0.181**
	(0.059)	(0.059)	(0.060)	(0.059)	(0.059)	(0.059)	(0.059)	(0.059)	(0.059)	(0.059)
Game Status	0.015	0.014	0.014	0.014	0.014	0.014	0.014	0.014	0.014	0.015
	(0.018)	(0.018)	(0.018)	(0.018)	(0.018)	(0.018)	(0.018)	(0.018)	(0.018)	(0.018)
Behavioral Feature	0.157***	-0.040	-0.008	-0.017	0.017	0.054*	0.064*	0.006	0.059*	0.075**
	(0.025)	(0.029)	(0.026)	(0.025)	(0.026)	(0.026)	(0.026)	(0.026)	(0.025)	(0.026)
T2*Game Role	-0.294*	-0.262*	-0.268*	-0.268*	-0.267*	-0.270*	-0.267*	-0.267*	-0.266*	-0.266*
	(0.115)	(0.117)	(0.117)	(0.117)	(0.117)	(0.117)	(0.117)	(0.117)	(0.117)	(0.116)
T3*Game Role	-0.499***	-0.519***	-0.516***	-0.514***	-0.516***	-0.514***	-0.516***	-0.515***	-0.512***	-0.499***
	(0.113)	(0.115)	(0.115)	(0.115)	(0.115)	(0.114)	(0.114)	(0.115)	(0.114)	(0.114)
Constant	3.305***	3.311***	3.289***	3.293***	3.290***	3.294***	3.293***	3.291***	3.280***	3.280***
	(0.076)	(0.079)	(0.078)	(0.077)	(0.077)	(0.077)	(0.077)	(0.077)	(0.077)	(0.077)
Observations	1,041	1,041	1,041	1,041	1,041	1,041	1,041	1,041	1,041	1,041

Log Likelihood	-1,198.795	-1,217.035	-1,217.936	-1,217.757	-1,217.769	-1,215.780	-1,214.857	-1,217.949	-1,215.272	-1,213.733

Note: + = p < .1, * = p < .05, ** = p < .01, *** = p < .001

Model Number	Behavioral Feature
1	Mean Vocal Loudness
2	Mean Vocal Pitch
3	Mean Head Pitch
4	Mean Head Yaw
5	Mean Head Roll
6	SD AU01
7	SD AU02
8	SD AU04
9	SD AU05
10	SD AU06

Supplementary Table 2: Mixed-effect regression results for nonverbal behaviors related to dominance (Model 11-20)

	Dependent variable:									
	Dominance									
	11	12	13	14	15	16	17	18	19	20
T2 (Round 1 and 2)	-0.040	-0.043	-0.041	-0.040	-0.047	-0.041	-0.041	-0.042	-0.036	-0.046
	(0.074)	(0.074)	(0.074)	(0.074)	(0.073)	(0.073)	(0.074)	(0.074)	(0.073)	(0.073)
T3 (After Round 2)	0.050	0.050	0.047	0.047	0.037	0.047	0.047	0.049	0.047	0.043
	(0.073)	(0.073)	(0.073)	(0.073)	(0.072)	(0.072)	(0.073)	(0.073)	(0.072)	(0.072)
Game Role (Spy = 1)	0.058	0.047	0.060	0.058	0.047	0.050	0.052	0.051	0.047	0.044
	(0.079)	(0.079)	(0.079)	(0.079)	(0.079)	(0.079)	(0.079)	(0.079)	(0.079)	(0.079)
Gender (Male = 1)	0.277***	0.258***	0.289***	0.297***	0.265***	0.202***	0.268***	0.258***	0.252***	0.297***
	(0.051)	(0.051)	(0.051)	(0.052)	(0.051)	(0.054)	(0.051)	(0.052)	(0.051)	(0.051)
Game Experience	0.071	0.066	0.071	0.073	0.072	0.082	0.074	0.075	0.090	0.067
	(0.057)	(0.057)	(0.057)	(0.057)	(0.056)	(0.057)	(0.057)	(0.057)	(0.057)	(0.056)
Native English Speaker	0.170**	0.166**	0.186**	0.173**	0.176**	0.193**	0.177**	0.179**	0.181**	0.161**
	(0.060)	(0.059)	(0.059)	(0.059)	(0.059)	(0.059)	(0.059)	(0.059)	(0.059)	(0.059)
Game Status	0.015	0.014	0.015	0.015	0.015	0.014	0.014	0.014	0.014	0.015
	(0.018)	(0.018)	(0.018)	(0.018)	(0.018)	(0.018)	(0.018)	(0.018)	(0.018)	(0.018)
Behavioral Feature	0.029	0.067**	0.072**	0.043^{+}	0.123***	0.106***	0.057*	0.059*	0.108***	0.126***
	(0.026)	(0.026)	(0.025)	(0.026)	(0.025)	(0.027)	(0.025)	(0.026)	(0.025)	(0.025)
T2*Game Role	-0.267*	-0.256*	-0.264*	-0.267*	-0.248*	-0.255*	-0.261*	-0.258*	-0.274*	-0.253*
	(0.117)	(0.117)	(0.116)	(0.117)	(0.116)	(0.116)	(0.117)	(0.117)	(0.116)	(0.116)
T3*Game Role	-0.513***	-0.512***	-0.506***	-0.506***	-0.479***	-0.502***	-0.503***	-0.508***	-0.505***	-0.496***
	(0.115)	(0.114)	(0.114)	(0.115)	(0.114)	(0.114)	(0.115)	(0.114)	(0.114)	(0.113)
Constant	3.294***	3.312***	3.282***	3.284***	3.301***	3.315***	3.296***	3.299***	3.295***	3.298***
	(0.077)	(0.077)	(0.077)	(0.077)	(0.077)	(0.078)	(0.077)	(0.077)	(0.077)	(0.076)
Observations	1,041	1,041	1,041	1,041	1,041	1,041	1,041	1,041	1,041	1,041

Log Likelihood	-1,217.329	-1,214.618	-1,214.009	-1,216.587	-1,206.101	-1,210.397	-1,215.445	-1,215.389	-1,209.063	-1,205.557

Note: + = p < .1, * = p < .05, ** = p < .01, *** = p < .001

Model Number	Behavioral Feature
11	SD AU07
12	SD AU09
13	SD AU10
14	SD AU12
15	SD AU14
16	SD AU15
17	SD AU17
18	SD AU20
19	SD AU23
20	SD AU25

Supplementary Table 3: Mixed-effect regression results for nonverbal behaviors related to dominance (Model 21-29)

	Dependent variable:								
	Dominance								
	21	22	23	24	25	26	27	28	29
T2 (Round 1 and 2)	-0.042	-0.038	-0.038	-0.043	-0.040	-0.037	0.098	-0.079	-0.035
	(0.074)	(0.074)	(0.074)	(0.073)	(0.074)	(0.074)	(0.119)	(0.067)	(0.073)
T3 (After Round 2)	0.046	0.053	0.055	0.053	0.054	0.054	0.157	0.005	0.039
	(0.072)	(0.073)	(0.073)	(0.072)	(0.073)	(0.073)	(0.117)	(0.066)	(0.072)
Game Role (Spy = 1)	0.039	0.054	0.062	0.050	0.053	0.054	0.226^{+}	0.062	0.049
	(0.079)	(0.079)	(0.079)	(0.079)	(0.079)	(0.079)	(0.137)	(0.072)	(0.078)
Gender (Male = 1)	0.253^{***}	0.301^{***}	0.254^{***}	0.209^{***}	0.271^{***}	0.270^{***}	0.276^{***}	0.173^{***}	0.324^{***}
	(0.051)	(0.051)	(0.053)	(0.053)	(0.051)	(0.051)	(0.077)	(0.047)	(0.051)
Game Experience	0.085	0.073	0.066	0.089	0.076	0.088	0.167^{+}	0.073	0.074
	(0.057)	(0.057)	(0.057)	(0.057)	(0.057)	(0.057)	(0.086)	(0.052)	(0.056)
Native English Speaker	0.173^{**}	0.182^{**}	0.178^{**}	0.180^{**}	0.176^{**}	0.175^{**}	0.254^{**}	0.134^{*}	0.176^{**}
	(0.059)	(0.059)	(0.059)	(0.059)	(0.059)	(0.059)	(0.085)	(0.055)	(0.058)
Game Status	0.014	0.015	0.014	0.014	0.014	0.014	0.041^{+}	0.015	0.015
	(0.018)	(0.018)	(0.018)	(0.018)	(0.018)	(0.018)	(0.024)	(0.016)	(0.018)
Behavioral Feature	0.085^{***}	0.075^{**}	-0.047^{+}	0.120^{***}	0.069^{**}	0.074^{**}	0.029	0.340^{***}	0.153^{***}
	(0.026)	(0.026)	(0.026)	(0.026)	(0.025)	(0.025)	(0.039)	(0.024)	(0.025)
T2*Game Role	-0.259^{*}	-0.273^{*}	-0.268^{*}	-0.253^{*}	-0.266^{*}	-0.273^{*}	-0.544^{**}	-0.158	-0.282^{*}
	(0.116)	(0.116)	(0.117)	(0.116)	(0.117)	(0.116)	(0.187)	(0.107)	(0.115)
T3*Game Role	-0.501^{***}	-0.520^{***}	-0.525^{***}	-0.519^{***}	-0.523^{***}	-0.522^{***}	-0.866^{***}	-0.397^{***}	-0.487^{***}
	(0.114)	(0.114)	(0.115)	(0.113)	(0.114)	(0.114)	(0.181)	(0.105)	(0.113)
Constant	3.303^{***}	3.280^{***}	3.302^{***}	3.314^{***}	3.294^{***}	3.288^{***}	3.008^{***}	3.353^{***}	3.273^{***}
	(0.077)	(0.077)	(0.077)	(0.077)	(0.077)	(0.077)	(0.124)	(0.073)	(0.077)

Observations	1,041	1,041	1,041	1,041	1,041	1,041	487	1,041	1,041
Log Likelihood	-1,212.570	-1,213.697	-1,216.353	-1,207.738	-1,214.276	-1,213.815	-587.230	-1,124.099	-1,199.900

Note: + = p < .1, * = p < .05, ** = p < .01, *** = p < .001

Model Number	Behavioral Feature
21	SD AU26
22	SD AU45
23	SD Vocal Pitch
24	SD Head Pitch
25	SD Head Yaw
26	SD Head Roll
27	Speaking Tempo (i.e., Word Count/Speaking Time)
28	Count of Turn-at-Talk
29	Mean Turn-at-Talk Duration

Supplementary Table 4: Mixed-effect regression results for nonverbal behaviors related to nervousness (Model 1-10)

	Dependent variable:									
	Nervousness									
	1	2	3	4	5	6	7	8	9	10
T2 (Round 1 and 2)	-0.153*	-0.151*	-0.151*	-0.152*	-0.150*	-0.150*	-0.150*	-0.150*	-0.150*	-0.150*
	(0.066)	(0.066)	(0.066)	(0.066)	(0.066)	(0.066)	(0.066)	(0.066)	(0.066)	(0.066)
T3 (After Round 2)	-0.207**	-0.208**	-0.209**	-0.208**	-0.208**	-0.208**	-0.208**	-0.208**	-0.207**	-0.208**
	(0.065)	(0.065)	(0.065)	(0.065)	(0.065)	(0.065)	(0.065)	(0.065)	(0.065)	(0.065)
Game Role (Spy = 1)	-0.052	-0.053	-0.054	-0.053	-0.053	-0.052	-0.052	-0.053	-0.053	-0.053
	(0.071)	(0.071)	(0.071)	(0.071)	(0.071)	(0.071)	(0.071)	(0.071)	(0.071)	(0.071)
Gender (Male = 1)	-0.108*	-0.107*	-0.093*	-0.104*	-0.111*	-0.112*	-0.110*	-0.111*	-0.114*	-0.110*
	(0.046)	(0.046)	(0.046)	(0.046)	(0.046)	(0.046)	(0.046)	(0.046)	(0.046)	(0.047)
Game Experience	-0.039	-0.038	-0.046	-0.035	-0.039	-0.036	-0.037	-0.037	-0.036	-0.036
	(0.051)	(0.051)	(0.051)	(0.051)	(0.051)	(0.051)	(0.051)	(0.051)	(0.051)	(0.051)
Native English Speaker	-0.103+	-0.110*	-0.108*	-0.114*	-0.112*	-0.111*	-0.108*	-0.111*	-0.113*	-0.111*
	(0.053)	(0.053)	(0.053)	(0.053)	(0.053)	(0.053)	(0.053)	(0.053)	(0.053)	(0.053)
Game Status	-0.010	-0.010	-0.010	-0.010	-0.010	-0.010	-0.010	-0.010	-0.010	-0.010
	(0.016)	(0.016)	(0.016)	(0.016)	(0.016)	(0.016)	(0.016)	(0.016)	(0.016)	(0.016)
Behavioral Feature	-0.038+	-0.013	-0.060**	0.033	-0.013	-0.006	-0.014	0.002	-0.022	0.0004
	(0.023)	(0.023)	(0.023)	(0.023)	(0.023)	(0.023)	(0.023)	(0.023)	(0.023)	(0.023)
T2*Game Role	0.142	0.136	0.135	0.138	0.135	0.135	0.135	0.136	0.134	0.135
	(0.105)	(0.105)	(0.104)	(0.105)	(0.105)	(0.105)	(0.105)	(0.105)	(0.105)	(0.105)
T3*Game Role	0.301**	0.304**	0.306**	0.304**	0.304**	0.303**	0.304**	0.305**	0.302**	0.305**
	(0.103)	(0.103)	(0.102)	(0.103)	(0.103)	(0.103)	(0.103)	(0.103)	(0.103)	(0.103)
Constant	2.662***	2.665***	2.663***	2.664***	2.668***	2.667***	2.665***	2.667***	2.669***	2.666***
	(0.069)	(0.069)	(0.069)	(0.069)	(0.070)	(0.070)	(0.069)	(0.070)	(0.069)	(0.070)
Observations	1,041	1,041	1,041	1,041	1,041	1,041	1,041	1,041	1,041	1,041

Log Likelihood	-1,102.796	-1,104.037	-1,100.791	-1,103.145	-1,104.031	-1,104.159	-1,104.013	-1,104.186	-1,103.722	-1,104.188

Note: + = p < .1, * = p < .05, ** = p < .01, *** = p < .001

Model Number	Behavioral Feature
1	Mean Vocal Loudness
2	SD AU01
3	SD AU02
4	SD AU04
5	SD AU05
6	SD AU06
7	SD AU07
8	SD AU09
9	SD AU10
10	SD AU12

Supplementary Table 5: Mixed-effect regression results for nonverbal behaviors related to nervousness (Model 11-20)

	Dependent variable:									
	Nervousness									
	11	12	13	14	15	16	17	18	19	20
T2 (Round 1 and 2)	-0.148*	-0.150*	-0.150*	-0.149*	-0.151*	-0.149*	-0.149*	-0.151*	-0.149*	-0.150*
	(0.066)	(0.066)	(0.066)	(0.066)	(0.066)	(0.066)	(0.066)	(0.066)	(0.066)	(0.066)
T3 (After Round 2)	-0.204**	-0.207**	-0.208**	-0.207**	-0.207**	-0.206**	-0.206**	-0.209**	-0.209**	-0.210**
	(0.065)	(0.065)	(0.065)	(0.065)	(0.065)	(0.065)	(0.065)	(0.065)	(0.065)	(0.065)
Game Role (Spy = 1)	-0.050	-0.049	-0.052	-0.050	-0.049	-0.049	-0.044	-0.050	-0.049	-0.049
	(0.071)	(0.071)	(0.071)	(0.071)	(0.071)	(0.071)	(0.071)	(0.071)	(0.071)	(0.071)
Gender (Male = 1)	-0.107*	-0.083+	-0.109*	-0.102*	-0.102*	-0.115*	-0.099*	-0.119**	-0.082+	-0.106*
	(0.046)	(0.049)	(0.046)	(0.046)	(0.046)	(0.046)	(0.046)	(0.046)	(0.048)	(0.046)
Game Experience	-0.037	-0.040	-0.037	-0.038	-0.042	-0.035	-0.043	-0.037	-0.044	-0.040
	(0.051)	(0.051)	(0.051)	(0.051)	(0.051)	(0.051)	(0.051)	(0.051)	(0.051)	(0.051)
Native English Speaker	-0.110*	-0.115*	-0.111*	-0.111*	-0.111*	-0.106*	-0.108*	-0.112*	-0.111*	-0.109*
	(0.053)	(0.053)	(0.053)	(0.053)	(0.053)	(0.053)	(0.053)	(0.053)	(0.053)	(0.053)
Game Status	-0.010	-0.010	-0.010	-0.010	-0.010	-0.010	-0.010	-0.010	-0.010	-0.010
	(0.016)	(0.016)	(0.016)	(0.016)	(0.016)	(0.016)	(0.016)	(0.016)	(0.016)	(0.016)
Behavioral Feature	-0.032	-0.038	-0.005	-0.024	-0.031	-0.032	-0.037	-0.031	-0.050*	-0.044+
	(0.023)	(0.024)	(0.023)	(0.023)	(0.023)	(0.023)	(0.023)	(0.023)	(0.024)	(0.023)
T2*Game Role	0.130	0.131	0.135	0.131	0.137	0.131	0.132	0.137	0.129	0.134
	(0.105)	(0.105)	(0.105)	(0.105)	(0.105)	(0.105)	(0.105)	(0.105)	(0.105)	(0.105)
T3*Game Role	0.295**	0.300**	0.303**	0.302**	0.302**	0.300**	0.298**	0.307**	0.306**	0.310**
	(0.103)	(0.103)	(0.103)	(0.103)	(0.103)	(0.103)	(0.103)	(0.103)	(0.103)	(0.103)
Constant	2.663***	2.657***	2.666***	2.663***	2.664***	2.664***	2.660***	2.670***	2.656***	2.664***
	(0.069)	(0.070)	(0.069)	(0.070)	(0.069)	(0.069)	(0.070)	(0.069)	(0.070)	(0.069)
Observations	1,041	1,041	1,041	1,041	1,041	1,041	1,041	1,041	1,041	1,041

Log Likelihood	-1,103.203	-1,102.981	-1,104.162	-1,103.651	-1,103.252	-1,103.204	-1,102.881	-1,103.304	-1,101.998	-1,102.339

Note: + = p < .1, * = p < .05, ** = p < .01, *** = p < .001

Model Number	Behavioral Feature
11	SD AU14
12	SD AU15
13	SD AU17
14	SD AU20
15	SD AU23
16	SD AU25
17	SD AU26
18	SD AU45
19	SD Head Pitch
20	SD Head Yaw

Supplementary Table 6: Mixed-effect regression results for nonverbal behaviors related to nervousness (Model 21-25)

	Dependent variable:				
	Nervousness				
	21	22	23	24	25
T2 (Round 1 and 2)	-0.152^{*}	-0.152^{*}	-0.143^{*}	-0.150^{*}	-0.151^{*}
	(0.066)	(0.066)	(0.066)	(0.066)	(0.066)
T3 (After Round 2)	-0.211^{**}	-0.204^{**}	-0.200^{**}	-0.210^{**}	-0.211^{**}
	(0.065)	(0.065)	(0.065)	(0.065)	(0.065)
Game Role (Spy = 1)	-0.048	-0.049	-0.053	-0.052	-0.055
	(0.071)	(0.071)	(0.071)	(0.071)	(0.071)
Gender (Male = 1)	-0.103^{*}	-0.126^{**}	-0.090^{+}	-0.073	-0.094^{*}
	(0.046)	(0.046)	(0.046)	(0.053)	(0.047)
Game Experience	-0.051	-0.037	-0.036	-0.034	-0.034
	(0.051)	(0.051)	(0.051)	(0.051)	(0.051)
Native English Speaker	-0.107^{*}	-0.109^{*}	-0.101^{+}	-0.109^{*}	-0.111^{*}
	(0.053)	(0.053)	(0.053)	(0.053)	(0.053)
Game Status	-0.010	-0.010	-0.010	-0.010	-0.010
	(0.016)	(0.016)	(0.016)	(0.016)	(0.016)
Behavioral Feature	-0.061^{**}	-0.052^{*}	-0.063^{**}	0.038	0.032
	(0.023)	(0.023)	(0.023)	(0.026)	(0.024)
T2*Game Role	0.140	0.140	0.115	0.130	0.135
	(0.104)	(0.105)	(0.105)	(0.105)	(0.105)
T3*Game Role	0.310^{**}	0.295^{**}	0.283^{**}	0.309^{**}	0.311^{**}
	(0.102)	(0.103)	(0.103)	(0.103)	(0.103)
Constant	2.667^{***}	2.671^{***}	2.653^{***}	2.647^{***}	2.659^{***}
	(0.069)	(0.069)	(0.069)	(0.071)	(0.070)
Observations	1,041	1,041	1,041	1,041	1,041
Log Likelihood	-1,100.630	-1,101.591	-1,100.470	-1,103.177	-1,103.274
Note:	+ = p < .1, * = p < .05, ** = p < .01, *** = p < .001				

Model Number	Behavioral Feature
21	SD Head Roll
22	Mean Turn-at-Talk Duration
23	Count of Turn-at-Talk
24	Mean Vocal Pitch
25	SD Vocal Pitch

Supplementary Table 7: Mixed-effect regression results for nonverbal behaviors related to trust (Model 1-10)

	Dependent variable:									
	Trustworthiness									
	1	2	3	4	5	6	7	8	9	10
T2 (Round 1 and 2)	-0.696***	-0.698***	-0.698***	-0.698***	-0.698***	-0.698***	-0.698***	-0.698***	-0.698***	-0.698***
	(0.072)	(0.072)	(0.072)	(0.072)	(0.072)	(0.072)	(0.072)	(0.072)	(0.072)	(0.072)
T3 (After Round 2)	-0.409***	-0.408***	-0.407***	-0.408***	-0.409***	-0.408***	-0.408***	-0.408***	-0.408***	-0.408***
	(0.070)	(0.070)	(0.070)	(0.070)	(0.070)	(0.070)	(0.070)	(0.070)	(0.070)	(0.070)
Game Role (Spy = 1)	-0.014	-0.014	-0.015	-0.014	-0.017	-0.015	-0.014	-0.014	-0.014	-0.014
	(0.077)	(0.077)	(0.077)	(0.077)	(0.077)	(0.077)	(0.077)	(0.077)	(0.077)	(0.077)
Gender (Male = 1)	-0.111*	-0.120*	-0.104*	-0.110*	-0.107*	-0.103*	-0.106*	-0.109*	-0.110*	-0.110*
	(0.049)	(0.056)	(0.049)	(0.049)	(0.049)	(0.049)	(0.049)	(0.049)	(0.049)	(0.049)
Game Experience	0.050	0.047	0.050	0.047	0.049	0.046	0.047	0.048	0.046	0.048
	(0.053)	(0.053)	(0.053)	(0.053)	(0.053)	(0.053)	(0.053)	(0.053)	(0.053)	(0.053)
Native English Speaker	-0.034	-0.029	-0.024	-0.028	-0.030	-0.027	-0.028	-0.029	-0.029	-0.029
	(0.055)	(0.055)	(0.055)	(0.055)	(0.055)	(0.055)	(0.055)	(0.055)	(0.055)	(0.055)
Game Status	0.107***	0.107***	0.107***	0.107***	0.107***	0.107***	0.107***	0.107***	0.107***	0.107***
	(0.016)	(0.016)	(0.016)	(0.016)	(0.016)	(0.016)	(0.016)	(0.016)	(0.016)	(0.016)
Behavioral Feature	0.026	-0.011	-0.021	-0.010	-0.020	-0.026	-0.010	0.003	-0.010	-0.003
	(0.025)	(0.028)	(0.025)	(0.025)	(0.025)	(0.025)	(0.025)	(0.025)	(0.025)	(0.025)
T2*Game Role	-0.305**	-0.299**	-0.301**	-0.301**	-0.301**	-0.300**	-0.301**	-0.301**	-0.301**	-0.301**
	(0.113)	(0.113)	(0.113)	(0.113)	(0.113)	(0.113)	(0.113)	(0.113)	(0.113)	(0.113)
T3*Game Role	-0.991***	-0.995***	-0.996***	-0.993***	-0.992***	-0.994***	-0.993***	-0.993***	-0.994***	-0.994***
	(0.111)	(0.111)	(0.111)	(0.111)	(0.111)	(0.111)	(0.111)	(0.111)	(0.111)	(0.111)
Constant	3.875***	3.878***	3.868***	3.874***	3.874***	3.871***	3.873***	3.873***	3.875***	3.873***
	(0.069)	(0.070)	(0.069)	(0.069)	(0.069)	(0.069)	(0.069)	(0.069)	(0.069)	(0.069)
Observations	1,041	1,041	1,041	1,041	1,041	1,041	1,041	1,041	1,041	1,041

Log Likelihood	-1,171.071	-1,171.562	-1,171.297	-1,171.560	-1,171.304	-1,171.087	-1,171.566	-1,171.631	-1,171.552	-1,171.633

Note: + = p < .1, * = p < .05, ** = p < .01, *** = p < .001

Model Number	Behavioral Feature
1	Mean Vocal Loudness
2	Mean Vocal Pitch
3	Mean Head Pitch
4	Mean Head Yaw
5	Mean Head Roll
6	SD AU01
7	SD AU02
8	SD AU04
9	SD AU05
10	SD AU06

Supplementary Table 8: Mixed-effect regression results for nonverbal behaviors related to trust (Model 11-20)

	Dependent variable:									
	Trustworthiness									
	11	12	13	14	15	16	17	18	19	20
T2 (Round 1 and 2)	-0.698***	-0.698***	-0.699***	-0.698***	-0.698***	-0.698***	-0.697***	-0.698***	-0.698***	-0.699***
	(0.072)	(0.072)	(0.071)	(0.072)	(0.072)	(0.072)	(0.072)	(0.072)	(0.072)	(0.072)
T3 (After Round 2)	-0.408***	-0.408***	-0.410***	-0.408***	-0.409***	-0.408***	-0.406***	-0.409***	-0.408***	-0.410***
	(0.070)	(0.070)	(0.070)	(0.070)	(0.070)	(0.070)	(0.070)	(0.070)	(0.070)	(0.070)
Game Role (Spy = 1)	-0.014	-0.015	-0.013	-0.014	-0.014	-0.014	-0.011	-0.015	-0.012	-0.017
	(0.077)	(0.077)	(0.077)	(0.077)	(0.077)	(0.077)	(0.077)	(0.077)	(0.077)	(0.077)
Gender (Male = 1)	-0.109*	-0.111*	-0.103*	-0.108*	-0.110*	-0.112*	-0.105*	-0.113*	-0.106*	-0.105*
	(0.049)	(0.049)	(0.049)	(0.050)	(0.049)	(0.052)	(0.049)	(0.049)	(0.049)	(0.049)
Game Experience	0.048	0.048	0.048	0.048	0.048	0.048	0.047	0.049	0.046	0.047
	(0.053)	(0.053)	(0.053)	(0.053)	(0.053)	(0.053)	(0.053)	(0.053)	(0.053)	(0.053)
Native English Speaker	-0.027	-0.029	-0.025	-0.029	-0.029	-0.028	-0.028	-0.028	-0.029	-0.032
	(0.055)	(0.055)	(0.055)	(0.055)	(0.055)	(0.055)	(0.055)	(0.055)	(0.055)	(0.055)
Game Status	0.107***	0.107***	0.107***	0.107***	0.107***	0.107***	0.107***	0.107***	0.107***	0.107***
	(0.016)	(0.016)	(0.016)	(0.016)	(0.016)	(0.016)	(0.016)	(0.016)	(0.016)	(0.016)
Behavioral Feature	-0.007	0.004	0.041+	0.003	0.008	0.004	-0.022	0.011	-0.014	0.028
	(0.025)	(0.025)	(0.025)	(0.025)	(0.025)	(0.026)	(0.025)	(0.025)	(0.025)	(0.025)
T2*Game Role	-0.301**	-0.300**	-0.299**	-0.301**	-0.300**	-0.301**	-0.303**	-0.299**	-0.300**	-0.298**
	(0.113)	(0.113)	(0.113)	(0.113)	(0.113)	(0.113)	(0.113)	(0.113)	(0.113)	(0.113)
T3*Game Role	-0.994***	-0.993***	-0.988***	-0.993***	-0.991***	-0.993***	-0.998***	-0.992***	-0.995***	-0.989***
	(0.111)	(0.111)	(0.111)	(0.111)	(0.111)	(0.111)	(0.111)	(0.111)	(0.111)	(0.111)
Constant	3.872***	3.874***	3.868***	3.872***	3.874***	3.874***	3.871***	3.875***	3.872***	3.875***
	(0.069)	(0.069)	(0.069)	(0.069)	(0.069)	(0.069)	(0.069)	(0.069)	(0.069)	(0.069)
Observations	1,041	1,041	1,041	1,041	1,041	1,041	1,041	1,041	1,041	1,041

Log Likelihood	-1,171.593	-1,171.623	-1,170.245	-1,171.630	-1,171.586	-1,171.628	-1,171.252	-1,171.548	-1,171.477	-1,170.986

Note: + = p < .1, * = p < .05, ** = p < .01, *** = p < .001

Model Number	Behavioral Feature
11	SD AU07
12	SD AU09
13	SD AU10
14	SD AU12
15	SD AU14
16	SD AU15
17	SD AU17
18	SD AU20
19	SD AU23
20	SD AU25

Supplementary Table 9: Mixed-effect regression results for nonverbal behaviors related to trust (Model 21-29)

	Dependent variable:								
	Trustworthiness								
	21	22	23	24	25	26	27	28	29
T2 (Round 1 and 2)	-0.698***	-0.698***	-0.698***	-0.697***	-0.698***	-0.698***	-0.757***	-0.704***	-0.696***
	(0.072)	(0.072)	(0.072)	(0.072)	(0.072)	(0.072)	(0.111)	(0.072)	(0.071)
T3 (After Round 2)	-0.409***	-0.409***	-0.408***	-0.408***	-0.408***	-0.408***	-0.307**	-0.415***	-0.412***
	(0.070)	(0.070)	(0.070)	(0.070)	(0.070)	(0.070)	(0.109)	(0.070)	(0.070)
Game Role (Spy = 1)	-0.018	-0.011	-0.014	-0.013	-0.015	-0.014	-0.017	-0.013	-0.017
	(0.077)	(0.077)	(0.077)	(0.077)	(0.077)	(0.077)	(0.127)	(0.077)	(0.077)
Gender (Male = 1)	-0.115*	-0.120*	-0.110*	-0.100*	-0.110*	-0.109*	-0.056	-0.125*	-0.094+
	(0.049)	(0.049)	(0.050)	(0.051)	(0.049)	(0.049)	(0.072)	(0.049)	(0.049)
Game Experience	0.051	0.047	0.048	0.046	0.049	0.048	0.116	0.048	0.049
	(0.053)	(0.053)	(0.053)	(0.053)	(0.053)	(0.053)	(0.080)	(0.053)	(0.053)
Native English Speaker	-0.030	-0.030	-0.029	-0.028	-0.029	-0.029	-0.040	-0.036	-0.030
	(0.055)	(0.055)	(0.055)	(0.055)	(0.055)	(0.055)	(0.079)	(0.055)	(0.055)
Game Status	0.107***	0.107***	0.107***	0.107***	0.107***	0.107***	0.146***	0.107***	0.107***
	(0.016)	(0.016)	(0.016)	(0.016)	(0.016)	(0.016)	(0.022)	(0.016)	(0.016)
Behavioral Feature	0.020	-0.039	-0.002	-0.016	0.013	0.00003	-0.090*	0.051*	0.054*
	(0.025)	(0.025)	(0.025)	(0.026)	(0.025)	(0.025)	(0.037)	(0.025)	(0.025)
T2*Game Role	-0.299**	-0.298**	-0.301**	-0.303**	-0.301**	-0.301**	-0.409*	-0.285*	-0.306**
	(0.113)	(0.113)	(0.113)	(0.113)	(0.113)	(0.113)	(0.174)	(0.113)	(0.113)
T3*Game Role	-0.990***	-0.991***	-0.994***	-0.993***	-0.995***	-0.993***	-1.335***	-0.976***	-0.983***
	(0.111)	(0.111)	(0.111)	(0.111)	(0.111)	(0.111)	(0.168)	(0.111)	(0.111)
Constant	3.876***	3.878***	3.873***	3.870***	3.874***	3.873***	3.781***	3.883***	3.868***
	(0.069)	(0.069)	(0.069)	(0.069)	(0.069)	(0.069)	(0.116)	(0.069)	(0.069)

Observations	1,041	1,041	1,041	1,041	1,041	1,041	487	1,041	1,041
Log Likelihood	-1,171.317	-1,170.407	-1,171.637	-1,171.454	-1,171.506	-1,171.639	-553.140	-1,169.606	-1,169.276

Note: + = p < .1, * = p < .05, ** = p < .01, *** = p < .001

Model Number	Behavioral Feature
21	SD AU26
22	SD AU45
23	SD Vocal Pitch
24	SD Head Pitch
25	SD Head Yaw
26	SD Head Roll
27	Speaking Tempo (i.e., Word Count/Speaking Time)
28	Count of Turn-at-Talk
29	Mean Turn-at-Talk Duration
